# Chromosome-scale assembly comparison of the Korean Reference Genome KOREF from PromethION and PacBio with Hi-C mapping information

**DOI:** 10.1093/gigascience/giz125

**Published:** 2019-12-03

**Authors:** Hui-Su Kim, Sungwon Jeon, Changjae Kim, Yeon Kyung Kim, Yun Sung Cho, Jungeun Kim, Asta Blazyte, Andrea Manica, Semin Lee, Jong Bhak

**Affiliations:** 1 KOGIC, Ulsan National Institute of Science and Technology (UNIST), UNIST-gil 50, Eonyang-eup, Ulju-gun, Ulsan 44919, Republic of Korea; 2 Department of Biomedical Engineering, School of Life Sciences, UNIST-gil 50, Eonyang-eup, Ulju-gun, UNIST, Ulsan 44919, Republic of Korea; 3 Clinomics Inc., UNIST-gil 50, Eonyang-eup, Ulju-gun, Ulsan 44919, Republic of Korea; 4 Personal Genomics Institute, Genome Research Foundation, Osong saengmyong1ro, Cheongju 28160, Republic of Korea; 5 Department of Zoology, Cambridge University, Downing street, Cambridge CB2 3EJ, UK

**Keywords:** Korean reference genome, KOREF, PromethION, Hi-C, nanopore sequencing, single-molecule sequencing

## Abstract

**Background:**

Long DNA reads produced by single-molecule and pore-based sequencers are more suitable for assembly and structural variation discovery than short-read DNA fragments. For *de novo* assembly, Pacific Biosciences (PacBio) and Oxford Nanopore Technologies (ONT) are the favorite options. However, PacBio's SMRT sequencing is expensive for a full human genome assembly and costs more than $40,000 US for 30× coverage as of 2019. ONT PromethION sequencing, on the other hand, is 1/12 the price of PacBio for the same coverage. This study aimed to compare the cost-effectiveness of ONT PromethION and PacBio's SMRT sequencing in relation to the quality.

**Findings:**

We performed whole-genome *de novo* assemblies and comparison to construct an improved version of KOREF, the Korean reference genome, using sequencing data produced by PromethION and PacBio. With PromethION, an assembly using sequenced reads with 64× coverage (193 Gb, 3 flowcell sequencing) resulted in 3,725 contigs with N50s of 16.7 Mb and a total genome length of 2.8 Gb. It was comparable to a KOREF assembly constructed using PacBio at 62× coverage (188 Gb, 2,695 contigs, and N50s of 17.9 Mb). When we applied Hi-C–derived long-range mapping data, an even higher quality assembly for the 64× coverage was achieved, resulting in 3,179 scaffolds with an N50 of 56.4 Mb.

**Conclusion:**

The pore-based PromethION approach provided a high-quality chromosome-scale human genome assembly at a low cost with long maximum contig and scaffold lengths and was more cost-effective than PacBio at comparable quality measurements.

## Data Description

Next-generation sequencing is a set of powerful sequencing technologies, and a recent trend in genomics is to use cost-effective long DNA reads for assembly and structural variation discovery using single-molecule sequencing methods. Oxford Nanopore Technologies (ONT) and Pacific Biosciences (PacBio) platforms have the advantages of a short run time and long read lengths over short fragmented reads by Illumina [1, 2]. Unfortunately, both methods share high base-calling error rates [2, 3]. However, bioinformatics pipelines for self-error correction and/or polishing sequences with short reads have become an effective option, and the overall accuracy of long-read–based assemblies is approaching what is required to be a viable option for personal reference genome construction [4]. Despite its excellent performance, PacBio's single-molecule real-time (SMRT) sequencing is expensive for the effective coverage required for a full human genome assembly, costing more than $40,000 US for 30× coverage (with 15 SMRT cells; from an estimated 6 Gb raw reads production per SMRT cell) as of 2019 [5–7]. On the other hand, the nanopore-based single-molecule, long-read platform PromethION from ONT is highly cost-effective at 1/12 the price of PacBio's for the same read amount, with an advantage of even longer average and maximum read lengths [8]. Although the 2 methods share some similarity, they are fundamentally different in that ONT uses a minimal amount of reagents with small form factor devices, and it can be a promising future technology for a broad scope of applications given its advantageous size and cost.

In this study, we performed benchmark tests of PromethION and PacBio with low and high coverages of sequencing data and investigated the advantages of pairing these long-read technologies with very long-range chromosome mapping information by Hi-C, using the already existing high-quality Korean reference genome, KOREF, as a benchmark [9].

### Whole-genome sequencing by ONT PromethION R9.4.1 platform

Human KOREF cell lines [10] were cultured at 37°C in 5% CO_2_ in RPMI-1640 medium with 10% heat-inactivated fetal bovine serum. DNA was extracted from cells using the DNeasy Blood & Tissue kit (Qiagen, Hilden, Germany). The KOREF cells (5 × 10^6^) were centrifuged at 300*g* for 5 min; the pelleted cells were suspended in 200 μL of phosphate-buffered saline and DNA was extracted according to the manufacturer's instructions. To preserve large-sized DNA and purify DNA fragments, we used Genomic DNA Clean & Concentrator kit (Zymo research, Irvine, CA, USA). The DNA quality and size were assessed by running 1 μL of purified DNA on the Bioanalyzer system (Agilent, Santa Clara, CA, USA). Concentration of DNA was assessed using the dsDNA BR assay on a Qubit fluorometer (Thermo Fisher Scientific, Carlsbad, CA, USA).

DNA repair (NEBNext formalin-fixed, paraffin-embedded [FFPE] DNA Repair Mix, NEB M6630) and end-prep (NEBNext End Repair/dA-tailing, NEB E7546) were performed using 1 μg human genomic DNA. The mixture of 1 μL DNA CS, 3.5 μL FFPE Repair Buffer, 2 μL FFPE DNA Repair Mix, 3.5 μL Ultra II End-prep reaction buffer, and 3 μL Ultra II End-prep enzyme mix was added to 47 μL DNA sample. The final mixture was incubated at 20°C for 5 min and then at 65°C for 5 min, cleaned up using 60 μL AMPure XP beads, incubated on Hula mixer for 5 min at room temperature, and washed twice with 200 μL fresh 70% ethanol. The pellet was allowed to dry for 30 s, and then DNA was eluted in 61 μL of nuclease-free water. An aliquot of 1 μL was quantified by Qubit to ensure that ≥1 μg DNA was retained.

Adaptor ligation was performed by adding 5 μL of Adaptor Mix (AMX, SQK-LSK109 Ligation Sequencing Kit 1D, Oxford Nanopore techonologies, Oxford, UK), 25 μL Ligation Buffer (LNB, SQK-LSK109), and 10 μL NEBNext Quick T4 DNA Ligase (NEB, E6056) to 60 μL bead cleaned-up DNA, followed by gentle mixing and incubation for 10 min at room temperature.

The adaptor-ligated DNA was cleaned up by adding 40 μL of AMPure XP beads, incubating for 5 min at room temperature, and resuspending the pellet twice in 250 μL L Fragment Buffer (LFB, SQK-LSK109). The purified ligated DNA was resuspended in 25 μL of Elution Buffer (ELB, SQK-LSK109), incubated for 10 min at room temperature, followed by pelleting the beads and transferring the supernatant (pre-sequencing mix) to a new Eppendorf Lobind tube. A 1-μL aliquot was quantified by Qubit to ensure that ≥500 ng DNA was retained.

To load the library, 75 μL of Sequencing Buffer (SQB, SQK-LSK109) was mixed with 51 μL of Loading Beads (LB, SQK-LSK109), and this mixture was added to 24 μL DNA library. This library was mixed by pipetting slowly, and 150 μL of sample was loaded through the inlet port.

### Whole-genome sequencing by PacBio Sequel platform

Genomic DNA was extracted from human KOREF blood samples using Qiagen Blood & Cell Culture DNA Kit (cat No. 13323, Qiagen, Hilden, Germany). A total of 5 μg of each sample was used as input for library preparation. The SMRTbell library was constructed using the SMRTbell® Express Template Preparation Kit (101-357-000, Pacific Biosciences, CA, USA). Using the BluePippin Size selection system we removed the small fragments for the large-insert library. After sequencing primer v4 was annealed to the SMRTbell template, DNA polymerase was bound to the complex (Sequel Binding kit 2.0, Pacific Biosciences, CA, USA). We purified the complex using AMPure Purification to remove excess primer and polymerase prior to sequencing. The SMRTbell library was sequenced using SMRT cells (PacBio) using Sequel Sequencing Kit v2.1 and 10 h movies were captured for each SMRT Cell 1M v2 using the Sequel (PacBio) sequencing platform.

### Short-read sequencing by Illumina HiSeq

Short paired-end raw reads using the Illumina HiSeq 2000 platform were acquired from a previous study, accession No. SRR2204706 [11].

### Hi-C chromosome conformation captured reads sequencing

Long-distance Hi-C chromosome conformation capture data were generated using the Arima-HiC kit (A160105 v01, San Diego, CA, USA), and double restriction enzymes were used for chromatin digestion. To prepare KOREF cell line samples for Hi-C analysis, cells were harvested and cross-linked as instructed by the manufacturer. One million cross-linked cells were used as input in the Hi-C protocol. Briefly, chromatin from cross-linked cells or nuclei was solubilized and then digested using restriction enzymes A1 and A2. The digested ends were then labeled using a biotinylated nucleotide, and ends were ligated to create ligation products. Ligation products were purified, fragmented, and selected by size using AMpure XP Beads. Biotinylated fragments were then enriched using Enrichment beads, and Illumina-compatible sequencing libraries were constructed on end repair, dA-tailing, and adaptor ligation using a modified workflow of the Hyper Prep kit (KAPA Biosystems, Inc.). The bead-bound library was then amplified, and amplicons were purified using AMpure XP beads and subjected to deep sequencing.

### Short and long sequence reads processing

A total of 144 Gb of short paired-end DNA raw reads were obtained from SRA2204706. Adaptor sequences were trimmed from sequenced raw reads using Trimmomatic v0.36 [12] (ILLUMINACLIP:2:30:10 LEADING:5 TRAILING:5 SLIDINGWINDOW:4:20 HEADCROP:15 MINLEN:60) (Trimmomatic, RRID:SCR_011848), and screening for vectors and microbial contaminants was performed using a customized database from Refseq. After pre-processing, a total of 137 Gb cleaned reads were obtained.

A total of 80.7 and 193 Gb raw reads (27× and 64× coverage) were obtained as a result of PromethION nanopore sequencing using 1 and 3 flowcells. Base-calling PromethION raw data was performed using Guppy v2.1.3 with the Transducer model. Removing adaptor sequences from the raw reads was performed using Porechop v0.2.4 (Porechop, RRID:SCR_016967) [13]. We also acquired 92.2 and 187.9 Gb raw reads from PacBio Sequel sequencing, resulting in 30× and 62× coverage (Table 1).

**Table 1:** Statistics of raw sequenced reads

Statistic	ONT PromethION R9.4.1		PacBio Sequel		Short-read Illumina HiSeq 2000
	27×	64×	30×	62×	
Number of reads	15,004,723	47,591,997	11,195,434	20,683,965	1,433,779,680
Total length of reads (bp)	80,770,821,288	193,027,803,978	92,229,416,062	187,914,740,184	144,811,747,680
N50 (bp)	12,736	9,190	13,426	14,568	101
Maximum contig length (bp)	774,322	1,160,324	65,865	169,910	101

### Long-read sequence–based *de novo* genome assemblies

*De novo* assemblies for the 27× and 64× PromethION raw reads were performed using wtdbg2 v2.3 (WTDBG, RRID:SCR_017225) [14]. To compare the accuracy, 2 sets of raw reads with 30× and 62× coverage of PacBio Sequel were also used employing the same assembler. Parameters for the assembler were set optimally for each sequencing platform with multiple trials [15]. For self-error correction with long reads, we generated consensus sequences using Racon v1.3.2 [16]. To improve the accuracy of assemblies, polishing consensus sequences with 48.2× coverage short reads was performed using Pilon v1.23 (Pilon, RRID:SCR_014731) [17]. To assess the completeness of the long-read genome assemblies, BUSCO v3.0.2 (BUSCO, RRID:SCR_015008) [18] with the default AUGUSTUS model for human was used to locate the presence and absence of 4,104 single-copy orthologous genes from mammalian OrthoDB v9.

For constructing chromosome-scale assemblies for the PromethION long-read data, map assembly with Hi-C reads was performed using SALSA2 v2.2 [19]. Duplicated Hi-C reads were removed using the clumpify.sh program from BBTools suite v38.32 (Bestus Bioinformaticus Tools, RRID:SCR_016968) [20]. Mapping Hi-C reads to the assembled genome was conducted using the pipeline provided by Arima-Genomics [21].

Long-read assemblies from 27× and 64× PromethION sequencing yielded total assembly sizes of 2,757 and 2,827 Mb, with contig N50s of 7.6 and 16.7 Mb, respectively (Table 2). Assemblies from PacBio sequencing at 30× and 62× coverage yielded the total assembly sizes of 2,800 and 2,815 Mb, with contig N50s of 11.1 and 17.9 Mb, respectively. Adding Hi-C reads to assemblies led to a 3.4- to 4.3-fold increase in the scaffold N50 lengths of PromethION (32.7 Mb for 27× coverage and 56.4 Mb for 64× coverage). For the PacBio assemblies, 2.2- to 3.3-fold increase was achieved for the scaffold N50 lengths (38.1 Mb for 30× coverage and 59.3 Mb for 62× coverage). The longest scaffold from both PromethION and PacBio assemblies with Hi-C was 2 times the length of the assemblies without Hi-C.

**Table 2:** Statistics of KOREF genome assemblies using ONT PromethION and PacBio Sequel sequencing

Statistic	ONT PromethION R9.4.1		PacBio Sequel	
	27× assembly	64× assembly	30× assembly	62× assembly
Contigs No.	3,262	3,725	2,443	2,695
Total length (bp)	2,757,297,803	2,827,624,042	2,800,962,512	2,815,311,932
N50 (bp)	7,655,153	16,706,773	11,137,362	17,931,968
Max contig length (bp)	60,569 695	88,903,341	50,101,007	77,816,513
Gap	0	0	0	0
GC content	40.82%	40.81%	40.90%	40.92%

### Comparison between PromethION and PacBio Assemblies

**Figure 1:**
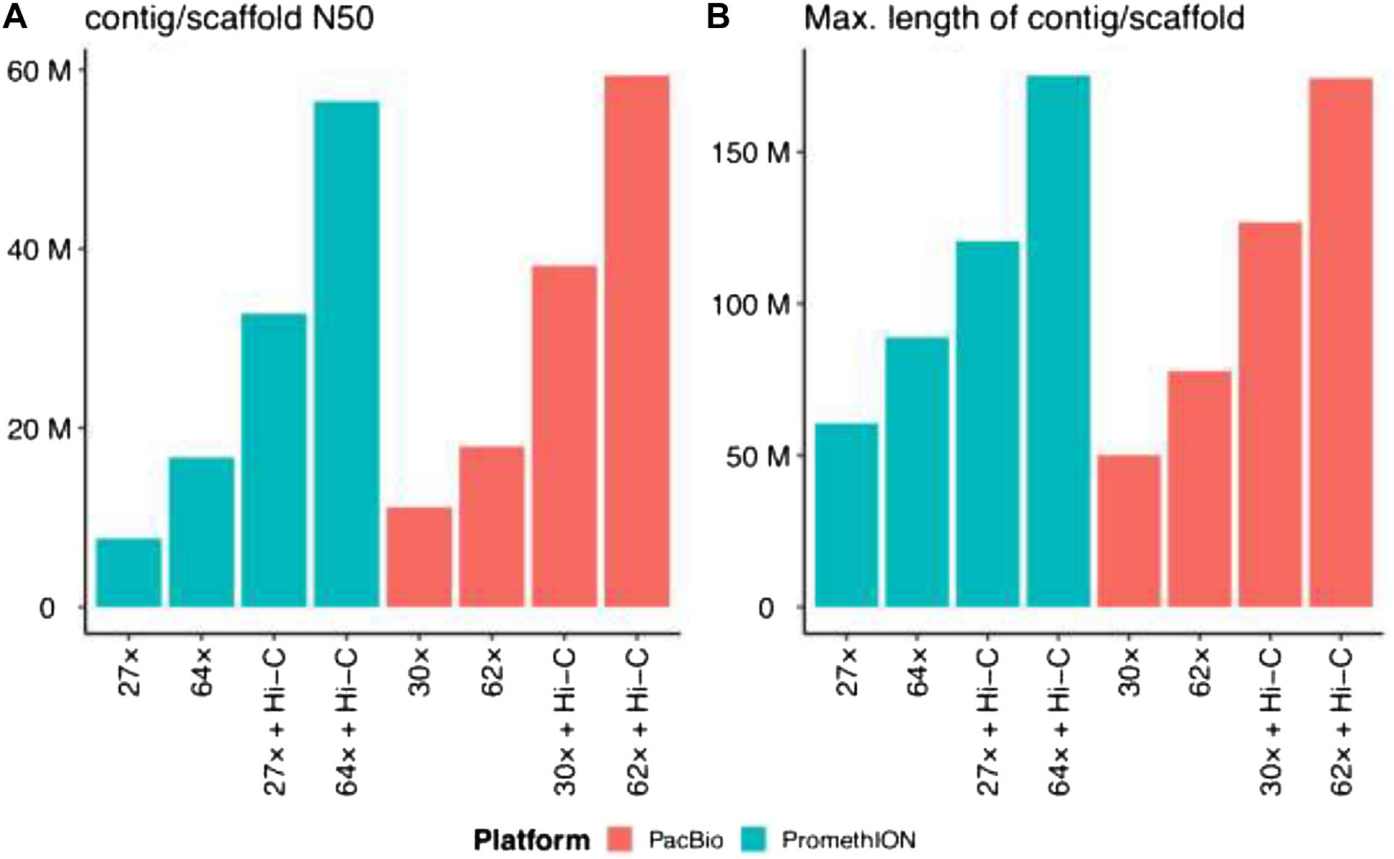
Comparison of (A) N50 lengths and (B) the longest contig or scaffold lengths for PromethION and PacBio assemblies of KOREF. Contig corresponds to assemblies without Hi-C data and scaffold corresponds to assemblies with Hi-C data.

The comparison between PromethION and PacBio assemblies without Hi-C mapping information using sequenced reads at 64× coverage showed comparable quality. In terms of N50, the PromethION assembly at 64× coverage yielded 1.5- and 0.93-fold longer N50s compared with the PacBio assemblies at 30× and 62× coverage, respectively (Fig. 1a). When we compared the longest contigs, the PromethION assembly at 64× coverage yielded 1.7- and 1.1-fold length increase compared with the PacBio assemblies at 30× and 62× coverage, respectively (Fig. 1b). Comparing the number of scaffolds, PacBio assembly at 30× coverage showed the fewest (2,443) compared with that of the PromethION assembly at 64× coverage (3,725) (Table 2).

When Hi-C mapping information was added to the assembly construction, the PromethION assembly at 64× coverage showed the best statistics as N50s of 56.4 Mb and the longest scaffold length of 175.2 Mb. The PromethION assembly at 27× coverage with Hi-C mapping information yielded 32.7 Mb for N50s, which was comparable to both 30× and 62× coverage PacBio assemblies with Hi-C; 0.85- and 0.55-fold for N50s, respectively (Table 3).

**Table 3:** Statistics of KOREF genome assemblies using ONT PromethION and PacBio Sequel sequencing with Hi-C mapping information

Statistic	ONT PromethION R9.4.1 assembly with Hi-C		PacBio Sequel assembly with Hi-C	
	27×	64×	30×	62×
Scaffolds No.	2,313	3,179	1,476	2,139
Total length (bp)	2,757,776,303	2,827,900,542	2,801,450,512	2,815,594,432
N50 (bp)	32,758,624	56,457,651	38,113,117	59,361,327
Maximum scaffold length (bp)	120,666,262	175,227,974	126,818,544	174,360,016
Gap	0.02%	0.01%	0.02%	0.01%
GC content	40.82%	40.81%	40.90%	40.90%

When we compared assessment results from BUSCO, all the assemblies that had been polished with short reads showed good quality; ∼92% completed orthologous genes with <1.1% completed and duplicated orthologous genes. Comparing the accuracy of the assemblies to the single assembly of KOREF (KOREF_S), which is the current standard, both showed ∼99.8% accuracy (Table 4). The accuracy comparison was performed using the assess_assembly program from Pomoxis [22].

**Table 4:** Statistics of KOREF genome assembly assessment using BUSCO and accuracy comparison

	ONT PromethION R9.4.1 (%)				PacBio Sequel (%)			
BUSCO assessment	27× assembly	64× assembly	27× assembly with Hi-C	64× assembly with Hi-C	30× assembly	62× assembly	30× assembly with Hi-C	62× assembly with Hi-C
Complete	92.5	92.7	92.6	94.0	93.8	93.9	93.8	93.5
Complete and single-copy	91.8	91.6	91.9	93.2	93.0	93.1	93.0	92.7
Complete and duplicated	0.7	1.1	0.7	0.8	0.8	0.8	0.8	0.8
Fragmented	3.1	3.7	3.2	3.1	3.0	2.9	3.0	3.1
Missing	4.4	3.6	4.2	2.9	3.2	3.2	3.2	3.4
Accuracy comparison*	99.78	99.73	99.78	99.73	99.83	99.79	99.86	99.80

* Compared with KOREF_S, the single assembly of KOREF.

## Conclusions

We generated high-quality assemblies of the Korean reference genome, KOREF, using ONT's PromethION long reads accompanied with Hi-C mapping information and compared them against PacBio sequencing and assemblies of the same sample. Comparing the results from the PromethION 64× sequencing to the PacBio 62× sequencing, we found that the former provided high contiguity and completeness at 1/12 the cost of PacBio. Results from just 27× PromethION sequencing combined with Hi-C mapping information were also comparable to the 30× coverage PacBio sequencing data. Therefore, to generate a chromosome-scale assembly with a long-read technology, at present, ONT's PromethION sequencing is a good alternative to PacBio's, owing to its quality and cost-effectiveness. Simple pore-based long-read sequencing has potential to dramatically improve sequencing and subsequent bioinformatics analysis for personal genome projects and cancer genome analyses where *de novo* assemblies are necessary for structural and copy number variations that cannot be detected easily by conventional short-read–only methods.

## Availability of Supporting Data and Materials

Raw long-read sequencing data from PromethION and PacBio are available at NCBI GenBank under the project accession number PRJNA549351. All genome assemblies of KOREF are available at the KOREF website (http://koref.net). Other supporting data and code are available from the *GigaScience* GigaDB repository [23].

## Abbreviations

bp: base pairs; BUSCO: Benchmarking Universal Single-Copy Orthologs; FFPE: formalin-fixed, paraffin-embedded; Gb: gigabase pairs; GC: guanine-cytosine; Mb: megabase pairs; NCBI: National Center for Biotechnology Information; ONT: Oxford Nanopore Technologies; PacBio: Pacific Biosciences; SMRT: single-molecule real-time.

## Competing interests

Y.S.C. is an employee, and J.B. is the CEO of Clinomics Inc. J.B. and Y.S.C. have an equity interest in the company. The other authors declare that they have no competing interests.

## Funding

This work was supported by U-K BRAND Research Fund (1.190007.01) of Ulsan National Institute of Science and Technology; Research Project Funded by Ulsan City Research Fund (1.190033.01) of Ulsan National Institute of Science and Technology and Clinomics internal funding for KOREF sequencing using a PromethION machine.

## Supplementary Material

giz125_GIGA-D-19-00240_Original_Submission

giz125_GIGA-D-19-00240_Revision_1

giz125_Response_to_Reviewer_Comments_Original_Submission

giz125_Reviewer_1_Report_Original_Submission Brock Peters, Ph.D. -- 7/15/2019 Reviewed

giz125_Reviewer_2_Report_Original_Submission Justin Zook -- 7/18/2019 Reviewed
