# Supplementary material for: Chromosome-scale assembly comparison of the Korean Reference Genome KOREF from PromethION and PacBio with Hi-C mapping information
Source: Gigascience. 2019 Dec 3;8(12):giz125. doi: 10.1093/gigascience/giz125 (PMC6889754; doi:10.1093/gigascience/giz125)
Supplement: giz125_GIGA-D-19-00240_Revision_1 [file giz125_giga-d-19-00240_revision_1.pdf]

GigaScience

Chromosome-scale assembly comparison of the Korean Reference Genome KOREF from PromethION and PacBio with Hi-C mapping information

--Manuscript Draft--

Manuscript Number:	GIGA-D-19-00240R1
Full Title:	Chromosome-scale assembly comparison of the Korean Reference Genome KOREF from PromethION and PacBio with Hi-C mapping information
Article Type:	Data Note
Funding Information:	Ulsan National Institute of Science and Technology (1.190007.01) — Dr. Jong Bhak<br>Ulsan National Institute of Science and Technology (1.190033.01) — Dr. Jong Bhak

Abstract:	Background<br>Long DNA reads produced by single molecule and pore-based sequencers are more suitable for assembly and structural variation discovery than short read DNA fragments. For de novo assembly, PacBio and Oxford Nanopore Technologies (ONT) are favorite options. However, PacBio’s SMRT sequencing is expensive for a full human genome assembly and costs over 40,000 USD for 30x coverage as of 2019. ONT PromethION sequencing, on the other hand, is one-twelfth the price of PacBio for the same coverage. This study aimed to compare the cost-effectiveness of ONT PromethION and PacBio’s SMRT sequencing in relation to the quality. Findings<br>We performed whole genome de novo assemblies and comparison to construct an improved version of KOREF, the Korean reference genome, using sequencing data produced by PromethION and PacBio. With PromethION, an assembly using sequenced reads with 64x coverage (193 Gb, 3 flowcell sequencing) resulted in 3,725 contigs with N50s of 16.7 Mbp and a total genome length of 2.8 Gbp. It was comparable to a KOREF assembly constructed using PacBio at 62x coverage (188 Gbp, 2,695 contigs and N50s of 17.9 Mbp). When we applied Hi-C-derived long-range mapping data, an even higher quality assembly for the 64x coverage was achieved, resulting in 3,179 scaffolds with an N50 of 56.4 Mbp.<br>Conclusion<br>The pore-based PromethION approach provides a good quality chromosome-scale human genome assembly at a low cost with long maximum contig and scaffold lengths and is more cost-effective than PacBio at comparable quality measurements.

Corresponding Author:	Jong Hwa Bhak, Ph.D.<br>UNIST<br>Ulsan, Ulsan KOREA, REPUBLIC OF
Corresponding Author Secondary Information:	
Corresponding Author's Institution:	UNIST
Corresponding Author's Secondary Institution:	
First Author:	Hui-Su Kim, Ph.D.
First Author Secondary Information:	
Order of Authors:	Hui-Su Kim, Ph.D.
	Sungwon Jeon, B.S.
	Changjae Kim, Ph.D.
	Yeon Kyung Kim, M.S.
	Yun Sung Cho, Ph.D.
	Jungeun Kim, Ph.D.

Powered by Editorial Manager® and ProduXion Manager® from Aries Systems Corporation

	Asta Blazyte, B.S.
	Andrea Manica, Ph.D.
	Semin Lee, Ph.D.
	Jong Bhak, Ph.D.
**Order of Authors Secondary Information:**	
**Response to Reviewers:**	Reviewer reports:<br><br>Reviewer #1:<br>Kim et al. generated multiple de novo assemblies of the Korean Reference Genome KOREF using nanopore and PacBio sequence data. These assemblies were polished with available short read data and further scaffolded with HiC data. These should be useful assemblies for the scientific community. All the data appears proper. I have a few issues that I think need to be resolved below:<br><br>1. Page 9, 7-16 talks about scaffold N50s, those same N50 lengths are referred to as contigs in the abstract. Which is it scaffolds or contigs? These two things are not the same.<br><br>You are right. To clarify: We used the concept of contigs and scaffolds as below. A contig is a contiguous genomic sequence without gaps in which the order of bases is known to a high confidence level. Mostly contigs are composed of overlapped reads from short or long read sequencing.<br>A scaffold is a portion of the genome sequence reconstructed from long range mated-pair short reads or the long-range mapping information such as Hi-C and BioNano data.<br>Therefore, the result from our assemblies with only long read sequencing was called as 'contigs' and the result from assemblies with Hi-C was denoted as 'scaffolds'.<br>To be clear, we changed the terms (contig or scaffold) of page 9, 7 – 16, accordingly.<br><br>2. Table 2 and Figure 1. Scaffolds and contigs are mixed here again. Why? This is confusing. These two things should be separated, they aren't the same. Perhaps with nanopore/Pacbio data only there's no difference between contig and scaffold length. However, the addition of Hi-C data would be expected to increase scaffold length but have little effect on contig length.<br><br>Good point. A new table with separated results from contigs and scaffolds is available (Table 2 and 3). We hope that this can be clear. We revised the legend of the Figure1.<br><br>Minor/interesting analyses:<br>1. Perhaps you are already doing this for another manuscript, but I think it would be interesting to compare between the different assemblies to see what regions are present/missing in nanopore versus PacBio. Was diploid assembly possible?<br><br>Interesting point. Thank you. Yes, we have assembled haplotype-resolved assemblies using the trio-binning method with KOREF_S (single individual reference). We are preparing another manuscript for these results and other in-depth analyses to see if PacBio and Promethion have segment differences in the assemblies. It is out of the scope of this paper. Although, it won't be included in the current paper, below is a preliminary alignment difference representation.<br><br>https://github.com/macarima/KOREF_PromethION_paper/blob/master/Supplementary_figures/images/Figure1-1.png<br><br>https://github.com/macarima/KOREF_PromethION_paper/blob/master/Supplementary_figures/images/Figure1-2.png<br><br>We also performed analysis of structural variation with assemblies. The patterns looked very similar (SVs from PromethION showed similar results to PacBio's). The

analysis was performed using Nucmer program from Mummer (https://github.com/mummer4/mummer) and Assemblytics v 1.0 (https://doi.org/10.1093/bioinformatics/btw369). These are not included in the revision.

https://github.com/macarima/KOREF_PromethION_paper/blob/master/Supplementary_figures/images/Figure2-1.png

https://github.com/macarima/KOREF_PromethION_paper/blob/master/Supplementary_figures/images/Figure2-2.png

2. If you try to call variants off of this assembly how well does it perform, perhaps since it's not diploid this won't work well.

It is another interesting research point. Yes, this is not a phased diploid assembly. It is supposed to be difficult to call variants efficiently without phasing information. We did not attempt to call.

Reviewer #2:
The authors compare assemblies from two different long read sequencing datasets (PacBio CLR and Oxford Nanopore Promethion) on the same Korean reference sample. These results are well-written, interesting, and useful to the community, though it would be good to add caveats to some of the conclusions given these quickly evolving technologies. In particular, it would be useful to discuss how these compare to the new PacBio CCS/HiFI assemblies. I also have a few suggestions below:

1. Abstract: "PacBio's SMRT sequencing is expensive for a full human genome assembly and costs over 40,000 USD for 30× coverage as of 2019"
- It's probably best to either make this less precise or say "early 2019" because my understanding is that the Sequel II now can give ~30x CCS coverage for $10-15k.

We agree. According to the reviewer's suggestion, we changed "2019" to "early 2019". Sequel II platform seems to show good performance with high accuracy (99.95% accuracy, https://doi.org/10.1101/635037) and is more cost-effective than Sequel. About contiguity, N50s between CCS and CLS assembly look almost the same. Sequel II can be benchmarked with PromethION R10 flowcell data which will be released soon (~Sep. 2019). PromethION R10 seems to have high accuracy as well (99.999% from the Oxford nanopore claim) like Sequel II (https://nanoporetech.com/about-us/news/new-r10-nanopore-released-early-access). When the new flowcells are released (R10), we will compare them and perhaps report in another manuscript.

2. Abstract: "The pore-based PromethION approach provides a good quality chromosome-scale human genome assembly at a low cost with long maximum contig and scaffold lengths and is more cost-effective than PacBio at comparable quality measurements"
- It would be good to discuss accuracy of the assemblies in addition to N50 metrics in the abstract, and ideally do a deeper analysis of accuracy.

It is an important point and of great interest, too. As it would take too much cross-validation and even more sequencing with short-reads, we cannot give a really high quality accuracy measurement here. However, we have performed alignment and calculated the accuracy with highly accurate KOREF_S assembly which has been assembled with both Illumina short reads and PacBio RS2 in Table 4 (Accuracy comparison section). We hope this can give some approximation for the current accuracy.
Again, when the new R10 Flowcell from ONT is released, we could do much more meaningful accuracy comparison while this manuscript focuses on cost-effectiveness of the two major single molecular long read sequencing platforms.

Powered by Editorial Manager® and ProduXion Manager® from Aries Systems Corporation

3. Page 9: "Long read assemblies from 27× and 64× PromethION sequencing yielded total assembly sizes of 2,757 Mbp and 2,827 Mbp, with scaffold N50s of 7.6 Mbp and 16.7 Mbp, respectively (Table 2). Assemblies from PacBio sequencing at 30× and 62× coverage yielded the total assembly sizes of 2,800 Mbp and 2,815 Mbp, with scaffold N50s of 11.1 Mbp and 17.9 Mbp, respectively."
-Before HiC, are the contig and scaffold N50's the same? If so, it would be good to state this explicitly

Thank you. It was not clear. For constructing chromosome scaled scaffolds, Hi-C map assembly was performed with 'contigs' from de novo assemblies with PromethION or PacBio reads. To make it clear, we have now divided 'Table 2' to Table 2 (statistics from contigs) and 3 (statistics from scaffolds).

4. Table 1: Are the PacBio read lengths the "sub-read" lengths, i.e. the lengths after breaking raw reads at the SMRT-bell adaptor sequences? If not, the sub-read statistics should also be given.

Thank you. Yes, they are the "sub-read" lengths.

5. It would be useful for the authors to discuss how this compares with the new PacBio CCS/HiFi method in these papers: https://doi.org/10.1101/635037and https://doi.org/10.1101/519025

Thank you for your advice. We have read the mentioned papers and have plans to produce CCS reads from Sequel II platform with the KOREF cell lines (KOREF_S has publically available cell-lines). In the near future, we will compare the PacBio CCS results with the one of PromethION R10.
For comparison between KOREF PromethION assemblies and CHM3 PacBio Sequel II assembly with the identical condition, we performed CHM3 assembly with wtdbg2 v2.3. Parameters which we used and other information could be found in the github page (https://github.com/macarima/KOREF_PromethION_paper). In terms of N50, the PacBio Sequel II assembly at 25× coverage yielded 1.9-fold and 0.8-fold longer N50s compared with the PromethION assemblies at 27× and 64× coverage, respectively. When we compared the longest contigs, the PacBio Sequel II assembly yielded 1.2-fold and 0.8-fold length increase compared with the PromethION assemblies at 27× and 64× coverage, respectively (Table S1).

https://github.com/macarima/KOREF_PromethION_paper/blob/master/Supplementary_figures/images/TableS1.png

6. I could not find the methods for base calling promethion data, which are important because the ONT base caller is quickly improving.

Good point. Thank you. Basecalling PromethION data was conducted using guppy v2.1.3 with the Transducer model. We added this information to the manuscript.

To reviewers,

All figures and tables are available on the github page (https://github.com/macarima/KOREF_PromethION_paper/tree/master/Supplementary_figures).
Thank you.

**Additional Information:**	
**Question**	**Response**
Are you submitting this manuscript to a	No

Powered by Editorial Manager® and ProduXion Manager® from Aries Systems Corporation

special series or article collection?	
**Experimental design and statistics**<br><br>Full details of the experimental design and statistical methods used should be given in the Methods section, as detailed in our Minimum Standards Reporting Checklist. Information essential to interpreting the data presented should be made available in the figure legends.<br><br>Have you included all the information requested in your manuscript?	Yes
**Resources**<br><br>A description of all resources used, including antibodies, cell lines, animals and software tools, with enough information to allow them to be uniquely identified, should be included in the Methods section. Authors are strongly encouraged to cite Research Resource Identifiers (RRIDs) for antibodies, model organisms and tools, where possible.<br><br>Have you included the information requested as detailed in our Minimum Standards Reporting Checklist?	Yes
**Availability of data and materials**<br><br>All datasets and code on which the conclusions of the paper rely must be either included in your submission or deposited in publicly available repositories (where available and ethically appropriate), referencing such data using a unique identifier in the references and in the "Availability of Data and Materials" section of your manuscript.<br><br>Have you have met the above requirement as detailed in our Minimum Standards Reporting Checklist?	Yes

Powered by Editorial Manager® and ProduXion Manager® from Aries Systems Corporation

Manuscript

Click here to view linked References

Click here to access/download;Manuscript;KOREF_PromethION.Draft.2019

Chromosome-scale assembly comparison of the Korean Reference Genome KOREF from PromethION and PacBio with Hi-C mapping information

Hui-Su Kim[1], Sungwon Jeon[1,2], Changjae Kim[1], Yeon Kyung Kim[1], Yun Sung Cho[3], Jungeun Kim[5], Asta Blazyte[1], Andrea Manica[4], Semin Lee[1,2]*, Jong Bhak[1,2,3,5]*

[1]KOGIC, Ulsan National Institute of Science and Technology (UNIST), Ulsan 44919, Republic of Korea

[2]Department of Biomedical Engineering, School of Life Sciences, UNIST, Ulsan 44919, Republic of Korea

[3]Clinomics Inc., Ulsan 44919, Republic of Korea

[4]Department of Zoology, Cambridge, University, Cambridge, UK

[5]Personal Genomics Institute, Genome Research Foundation, Cheongju 28160, Republic of Korea

***Correspondence author:**

Name: Jong Bhak, Ph.D.

Address: #110-303, Ulsan National Institute of Science and Technology, UNIST-gil 50, Eonyang-eup, Ulju-gun, Ulsan 44919, Republic of Korea

Phone: (+82) 10-4644-6754

Email: jongbhak@genomics.org, ORCID: 0000-0002-4228-1299

Name: Semin Lee, Ph.D.

Address: #110-302, Ulsan National Institute of Science and Technology, UNIST-gil 50, Eonyang-eup, Ulju-gun, Ulsan 44919, Republic of Korea

Phone: (+82) 52-217-2663

Email: seminlee@gmail.com, ORCID: 0000-0002-9015-6046

Other ORCID IDs:
Andrea Manica: 0000-0003-1895-450X

Abstract

Background: Long DNA reads produced by single molecule and pore-based sequencers are more suitable for assembly and structural variation discovery than short read DNA fragments. For *de novo* assembly, PacBio and Oxford Nanopore Technologies (ONT) are the favorite options. However, PacBio's SMRT sequencing is expensive for a full human genome assembly and costs over 40,000 USD for 30× coverage as of 2019. ONT PromethION sequencing, on the other hand, is one-twelfth the price of PacBio for the same coverage. This study aimed to compare the cost-effectiveness of ONT PromethION and PacBio's SMRT sequencing in relation to the quality.

Findings: We performed whole genome *de novo* assemblies and comparison to construct an improved version of KOREF, the Korean reference genome, using sequencing data produced by PromethION and PacBio. With PromethION, an assembly using sequenced reads with 64× coverage (193 Gb, 3 flowcell sequencing) resulted in 3,725 contigs with N50s of 16.7 Mbp and a total genome length of 2.8 Gbp. It was comparable to a KOREF assembly constructed using PacBio at 62× coverage (188 Gbp, 2,695 contigs and N50s of 17.9 Mbp). When we applied Hi-C-derived long-range mapping data, an even higher quality assembly for the 64× coverage was achieved, resulting in 3,179 scaffolds with an N50 of 56.4 Mbp.

Conclusion: The pore-based PromethION approach provides a good quality chromosome-scale human genome assembly at a low cost with long maximum contig and scaffold lengths and is more cost-effective than PacBio at comparable quality measurements.

Keywords: Korean reference genome; KOREF, PromethION; Hi-C; nanopore sequencing, single molecule sequencing

Data Description

Next-generation sequencing (NGS) is a set of powerful sequencing technologies and a recent trend in genomics is to use cost-effective long DNA reads for assembly and structural variation discovery using single molecule sequencing methods. Oxford Nanopore Technologies (ONT) and PacBio platforms have advantages of a short run time and long read lengths over short fragmented reads by Illumina [1, 2]. Unfortunately, both methods share high base-calling error rates [3, 4]. However, bioinformatics pipelines for self-error correction and/or polishing sequences with short reads have become an effective option, and the overall accuracy of long read based assemblies is approaching what is required to be a viable option for personal reference genome construction [5]. Despite its excellent performance, PacBio's SMRT sequencing is expensive for the effective coverage required for a full human genome assembly, costing over 40,000 USD for 30× coverage (with 15 SMRT cells; from an estimated 6 Gbp raw reads production per SMRT cell) as of 2019 [6, 7, 8]. On the other hand, the nanopore based single molecule, long read platform, PromethION from ONT is highly cost-effective at one-twelfth the price of PacBio's for the same read amount, with an advantage of even longer average and maximum read lengths [9]. Although the two methods share some similarity, they are fundamentally different in that ONT uses a minimal amount of reagents with small form factor devices, and can be a promising future technology for a very broad scope of applications given its advantageous size and cost.

In this study, we performed benchmark tests of PromethION and PacBio with low and high coverages of sequencing data and investigated the advantages of pairing these long read technologies with very long-range chromosome mapping information by Hi-C, using the already existing high-quality Korean reference genome, KOREF, as a benchmark [10].

Whole genome sequencing by ONT PromethION R9.4.1 platform

Human KOREF cell lines (http://koref.net) were cultured at 37°C in 5% CO_2 in RPMI-1640 medium with 10% heat-inactivated fetal bovine serum. DNA was extracted from cells using the DNeasy Blood & Tissue kit (Qiagen). The KOREF cells (5 x 10^6) were centrifuged at 300 *g* for 5 min; the pelleted cells were suspended in 200 µL of PBS and DNA was extracted according to the manufacturer's instructions. To preserve large-sized DNA and purify DNA fragments, we used Genomic DNA Clean & Concentrator kit (Zymo). The DNA quality and size were assessed by running 1 µL of purified DNA on the Bioanalyzer system (Agilent). Concentration of DNA was assessed using the dsDNA BR assay on a Qubit fluorometer (Thermo Fisher).

DNA repair (NEBNext FFPE DNA Repair Mix, NEB M6630) and end-prep (NEBNext End Repair/dA-tailing, NEB E7546) were performed using 1 µg human genomic DNA. The mixture of 1 µL DNA CS, 3.5 µL FFPE Repair Buffer, 2 µL FFPE DNA Repair Mix, 3.5 µL Ultra II End-prep reaction buffer, and 3 µL Ultra II End-prep enzyme mix was added to 47 µL DNA sample. The final mixture was incubated at 20°C for 5 min and then at 65°C for 5 min, cleaned up using 60 µL AMPure XP beads, incubated on Hula mixer for 5 min at room temperature, and washed twice with 200 µL fresh 70% ethanol. The pellet was allowed to dry for 30 s, and then DNA was eluted in 61 µL of nuclease-free water. An aliquot of 1 µL was quantified by Qubit to ensure ≥ 1 µg DNA was retained.

Adaptor ligation was performed by adding 5 µL of Adaptor Mix (AMX, SQK-LSK109 Ligation Sequencing Kit 1D, Oxford Nanopore Technologies (ONT)), 25 µL Ligation Buffer (LNB, SQK-LSK109), and 10 µL NEBNext Quick T4 DNA Ligase (NEB, E6056) to 60 µL bead cleaned-up DNA, followed by gentle mixing and incubation for 10 min at room temperature.

The adaptor-ligated DNA was cleaned up by adding 40 μL of AMPure XP beads, incubating for 5 min at room temperature and re-suspending the pellet twice in 250 μL L Fragment Buffer (LFB, SQK-LSK109). The purified ligated DNA was re-suspended in 25 μL of Elution Buffer (ELB, SQK-LSK109), incubated for 10 min at room temperature, followed by pelleting the beads, and transferring the supernatant (pre-sequencing mix or PSM) to a new Eppendorf Lobind tube. A 1-μL aliquot was quantified by Qubit to ensure ≥ 500 ng DNA was retained.

To load the library, 75 μL of Sequencing Buffer (SQB, SQK-LSK109) was mixed with 51 μL of Loading Beads (LB, SQK-LSK109) and this mixture was added to 24 μL DNA library. This library was mixed by pipetting slowly and 150 μL of sample was loaded through the inlet port.

Whole genome sequencing by PacBio Sequel platform

Genomic DNA was extracted from human KOREF blood samples using QIAGEN Blood & Cell Culture DNA Kit (cat no 13323). A total of 5 μg of each sample was used as input for library preparation. The SMRTbell library was constructed using SMRTbell® Express Template Preparation Kit (101-357-000). Using the BluePippin Size selection system we removed the small fragments for large-insert library. After sequencing primer v4 was annealed to the SMRTbell template, DNA polymerase was bound to the complex (Sequel Binding kit 2.0). We purified the complex using AMPure Purification to remove excess primer and polymerase prior to sequencing. The SMRTbell library was sequenced using SMRT cells (Pacific Biosciences) using Sequel Sequencing Kit v2.1 and 10 h movies were captured for each SMRT Cell 1M v2 using the Sequel (Pacific Biosciences) sequencing platform.

Short read sequencing by Illumina HiSeq

Short paired-end raw reads using Illumina HiSeq 2000 platform were acquired from a previous study, accession no. SRR2204706 (ftp://ftp.sra.ebi.ac.uk/vol1/srr/SRR220/006/SRR2204706).

Hi-C chromosome conformation captured reads sequencing

Long distance Hi-C chromosome conformation capture data were generated using the Arima-HiC kit (A160105 v01), and double restriction enzymes were used for chromatin digestion. To prepare KOREF cell line samples for Hi-C analysis, cells were harvested and cross-linked as instructed by the manufacturer. One million cross-linked cells were used as input in the Hi-C protocol. Briefly, chromatin from cross-linked cells or nuclei was solubilized, and then digested using restriction enzymes A1 and A2. The digested ends were then labeled using a biotinylated nucleotide, and ends were ligated to create ligation products. Ligation products were purified, fragmented, and selected by size using AMpure XP Beads. Biotinylated fragments were then enriched using Enrichment beads, and Illumina-compatible sequencing libraries were constructed on End Repair, dA-tailing, and Adaptor Ligation using a modified workflow of the Hyper Prep kit (KAPA Biosystems, Inc.). The bead-bound library was then amplified, and amplicons were purified using AMpure XP beads and subjected to deep sequencing.

Short and long sequence reads processing

A total of 144 Gbp of short paired-end DNA raw reads were obtained from SRA2204706. Adapter sequences were trimmed from sequenced raw reads using Trimmomatic v0.36[11]

(ILLUMINACLIP:2:30:10 LEADING:5 TRAILING:5 SLIDINGWINDOW:4:20 HEADCROP:15 MINLEN:60) (Trimmomatic, RRID:SCR_011848), and screening for vectors and microbial contaminants were performed using customized database from Refseq. After preprocessing, a total of 137 Gbp cleaned reads were obtained.

A total of 80.7 Gbp and 193 Gbp raw reads (27× and 64× coverage) were obtained as a result of PromethION nanopore sequencing using one and three flowcells. Basecalling PromethION raw data was performed using Guppy v2.1.3 with the Transducer model. Removing adapter sequences from the raw reads was performed using Porechop v0.2.4 (Porechop, RRID:SCR_016967) [12]. We also acquired 92.2 Gbp and 187.9 Gbp raw reads from PacBio Sequel sequencing resulting in 30× and 62× coverage (Table 1).

Long-read sequence based *de novo* genome assemblies

De novo assemblies for the 27× and 64× PromethION raw reads were performed using wtdbg2 v2.3 (WTDBG, RRID:SCR_017225) [13]. To compare the accuracy, two sets of raw reads with 30× and 62× coverage of PacBio Sequel were also used employing the same assembler. Parameters for the assembler were set optimally for each sequencing platform with multiple trials (https://github.com/macarima/KOREF_PromethION_paper). For self-error correction with long reads, we generated consensus sequences using Racon v1.3.2 [14]. To improve the accuracy of assemblies, polishing consensus sequences with 48.2× coverage short reads was performed using Pilon v1.23 (Pilon, RRID:SCR_014731) [15]. To assess the completeness of the long-read genome assemblies, BUSCO v3.0.2 (BUSCO, RRID:SCR_015008) [16] with the default AUGUSTUS model for human was used to locate the presence and absence of 4,104 single copy orthologous genes from mammalian OrthoDB v9.

For constructing chromosome-scale assemblies for the PromethION long-reads data, map assembly with Hi-C reads was performed using SALSA2 v2.2 [17]. Duplicated Hi-C reads were removed using clumpify.sh program from BBTools suite v38.32 (Bestus Bioinformaticus Tools, RRID:SCR_016968) [18]. Mapping Hi-C reads to the assembled genome was conducted using the pipeline provided by Arima-Genomics (https://github.com/ArimaGenomics/mapping_pipeline).

Long read assemblies from 27× and 64× PromethION sequencing yielded total assembly sizes of 2,757 Mbp and 2,827 Mbp, with contig N50s of 7.6 Mbp and 16.7 Mbp, respectively (Table 2). Assemblies from PacBio sequencing at 30× and 62× coverage yielded the total assembly sizes of 2,800 Mbp and 2,815 Mbp, with contig N50s of 11.1 Mbp and 17.9 Mbp, respectively. Adding Hi-C reads to assemblies led to 3.4- to 4.3-fold increase in the scaffold N50 lengths of PromethION (32.7 Mbp for 27× coverage and 56.4 Mbp for 64× coverage). For the PacBio assemblies, 2.2- to 3.3-fold increase was achieved for the scaffold N50 lengths (38.1 Mbp for 30× coverage and 59.3 Mbp for 62× coverage). The longest scaffold from both PromethION and PacBio assemblies with Hi-C was two times the length of the assemblies without Hi-C.

Comparison between PromethION and PacBio assemblies

The comparison between PromethION and PacBio assemblies without Hi-C mapping information using sequenced reads at 64× coverage showed comparable quality. In terms of N50, the PromethION assembly at 64× coverage yielded 1.5-fold and 0.93-fold longer N50s compared with the PacBio assemblies at 30× and 62× coverage, respectively (Figure 1a). When we compared the longest contigs, the PromethION assembly at 64× coverage yielded 1.7-fold and 1.1-fold length increase compared with the PacBio assemblies at 30× and 62× coverage,

respectively (Figure 1b). Comparing the number of scaffolds, PacBio assembly at 30× coverage showed the fewest (2,443) compared with that of PromethION assembly at 64× coverage (3,725) (Table 2).

When Hi-C mapping information was added to the assembly construction, the PromethION assembly at 64× coverage showed the best statistics as N50s of 56.4 Mbp and the longest scaffold length of 175.2 Mbp. The PromethION assembly at 27× coverage with Hi-C mapping information yielded 32.7 Mbp for N50s, which was comparable to both 30× and 62× coverage PacBio assemblies with Hi-C; 0.85-fold and 0.55-fold for N50s, respectively (Table 3).

When we compared assessment results from BUSCO, all the assemblies that had been polished with short reads showed good quality; around 92% completed orthologous genes with less than 1.1% completed and duplicated orthologous genes. Comparing the accuracy of the assemblies to the single assembly of KOREF (KOREF_S), which is the current standard, both showed around 99.8% accuracy (Table 4). The accuracy comparison was performed using the assess_assembly program from Pomoxis [19].

Conclusions

We generated high-quality assemblies of the Korean reference genome, KOREF, using ONT’s PromethION long-reads accompanied with Hi-C mapping information and compared them against PacBio sequencing and assemblies of the same sample. Comparing the results from the PromethION 64× sequencing to the PacBio 62× sequencing, we found that the former provided high contiguity and completeness at one-twelfth the cost of PacBio. Results from just 27× PromethION sequencing combined with Hi-C mapping information were also comparable to

the 30× coverage PacBio sequencing data. Therefore, to generate a chromosome-scale assembly with a long-read technology, at present, the ONT's PromethION sequencing is a good alternative to PacBio's, owing to its quality and cost-effectiveness. Simple pore-based long read sequencing has potential to dramatically improve sequencing and subsequent bioinformatics analysis for personal genome projects and cancer genome analyses where *de novo* assemblies are necessary for structural and copy number variations that cannot be detected easily by conventional short read only methods.

Availability of supporting data

Raw long-read sequencing data from PromethION and PacBio is available at NCBI genbank under the project accession number PRJNA549351. All genome assemblies of KOREF are available at KOREF website (http://koref.net). Other supporting data and code is available from the GigaScience GigaDB repository[].

Abbreviations

BUSCO: Benchmarking Universal Single-Copy Orthologs; PacBio: Pacific Biosciences; SMRT: single-molecule real-time

Competing interests

Y.S.C. is an employee, and J.B. is the CEO of Clinomics Inc. J.B. and Y.S.C. have an equity interest in the company. All other coauthors have no conflicts of interest to declare.

Funding

This work was supported by U-K BRAND Research Fund (1.190007.01) of UNIST; Research Project Funded by Ulsan City Research Fund (1.190033.01) of UNIST and Clinomics internal funding for KOREF sequencing using a PromethION machine.

Figure Legends

Figure 1. Comparison of (A) N50 lengths and (B) the longest contig or scaffold lengths for PromethION and PacBio assemblies of KOREF. Contig corresponds to assemblies without Hi-C data and scaffold corresponds to assemblies with Hi-C data.

References

1. Mccarthy A. Third generation DNA sequencing: Pacific biosciences' single molecule real time technology. 2010 Jul 30;17(7):675-6. doi:10.1016/j.chembiol.2010.07.004.
2. Laver T, Harrison J, O'Neill PA, Moore K, Farbos A, Paszkiewicz K, et al. Assessing the performance of the Oxford Nanopore Technologies MinION. Biomol Detect Quantif. Elsevier; 2015;3:1–8.
3. Chin C-S, Alexander DH, Marks P, Klammer AA, Drake J, Heiner C, et al. Nonhybrid, finished microbial genome assemblies from long-read SMRT sequencing data. Nat Methods. 2013 Jun;10(6):563-9. doi: 10.1038/nmeth.2474.
4. Laver T, Harrison J, O'Neill PA, Moore K, Farbos A, Paszkiewicz K, et al. Assessing the performance of the Oxford Nanopore Technologies MinION. Biomol
5. Detect Quantif. 2015 Mar;3:1-8.
6. Fu S, Wang A, Au KF. A comparative evaluation of hybrid error correction methods for error-prone long reads. Genome Biol. 2019 Feb 4;20(1):26. doi: 10.1186/s13059-018-1605-z.
7. Desk R. Review article. Toenail onychomycosis: an important global disease burden.. N. Engl. J. Med. 2005. p. 1–4.
8. University of Washington PacBio Sequencing Services. https://pacbio.gs.washington.edu/
9. UC Davis Genome Center: https://dnatech.genomecenter.ucdavis.edu/prices/
10. Nanopore tech. https://nanoporetech.com/products/comparison/
11. Cho YS, Kim H, Kim HM, Jho S, Jun JH, Lee YJ, et al. Corrigendum: An ethnically relevant consensus Korean reference genome is a step towards personal reference genomes. Nat Commun. 2017 Oct 9;8:16168. doi: 10.1038/ncomms16168.

12. Bolger AM, Lohse M, Usadel B. Trimmomatic: A flexible trimmer for Illumina sequence data. Bioinformatics. 2014 Aug 1;30(15):2114-20. doi:10.1093/bioinformatics/btu170.
13. Porechop, adapter trimmer for Oxford Nanopore reads. https://github.com/rrwick/Porechop
14. Ruan J, Li H. Fast and accurate long-read assembly with wtdbg2. bioRxiv. 2019;530972. https://doi.org/10.1101/530972
15. Racon, ultrafast consensus module for raw de novo genome assembly of long uncorrected reads. https://github.com/isovic/racon
16. Walker BJ, Abeel T, Shea T, Priest M, Abouelliel A, Sakthikumar S, et al. Pilon: An integrated tool for comprehensive microbial variant detection and genome assembly improvement. PLoS One. 2014 Nov19;9(11):e112963. doi: 10.1371/journal.pone.0112963.
17. Simão FA, Waterhouse RM, Ioannidis P, Kriventseva E V., Zdobnov EM. BUSCO: Assessing genome assembly and annotation completeness with single-copy orthologs. Bioinformatics. 2015 Oct 1;31(19):3210-2. doi: 10.1093/bioinformatics/btv351.
18. Ghurye J, Pop M, Koren S, Bickhart D, Chin CS. Scaffolding of long read assemblies using long range contact information. BMC Genomics. 2017 Jul 12;18(1):527. doi: 10.1186/s12864-017-3879-z.
19. BBMap, short read aligner and other bioinformatics tools. https://sourceforge.net/projects/bbmap/
20. Pomoxis, bioinformatics tools for nanopore research. https://nanoporetech.github.io/pomoxis/
21. Kim H; Jeon S; Kim C; Kim YK; Cho YS; Kim J; Blazyte A; Manica A; Lee S; Bhak J (2019): Supporting data for "Chromosome-scale assembly comparison of the Korean

Reference Genome KOREF from PromethION and PacBio with Hi-C mapping information" GigaScience Database. http://dx.doi.org/10.5524/100654

Table 1. Statistics of raw sequenced reads

	ONT PromethION R9.4.1		PacBio Sequel		Short read
	**27×**	**64×**	**30×**	**62×**	**Illumina HiSeq 2000**
**Number of reads**	15,004,723	47,591,997	11,195,434	20,683,965	1,433,779,680
**Total length of reads (bp)**	80,770,821,288	193,027,803,978	92,229,416,062	187,914,740,184	144,811,747,680
**N50 (bp)**	12,736	9,190	13,426	14,568	101
**Max contig length (bp)**	774,322	1,160,324	65,865	169,910	101

Table 2. Statistics of KOREF genome assemblies using ONT PromethION and PacBio Sequel sequencing

	ONT PromethION R9.4.1		PacBio Sequel	
	**27× assembly**	**64× assembly**	**30× assembly**	**62× assembly**
**Contigs No.**	3,262	3,725	2,443	2,695
**Total length (bp)**	2,757,297,803	2,827,624,042	2,800,962,512	2,815,311,932
**N50 (bp)**	7,655,153	16,706,773	11,137,362	17,931,968
**Max contig length (bp)**	60,569,695	88,903,341	50,101,007	77,816,513
**Gap**	0.00%	0.00%	0.00%	0.00%
**GC content**	40.82%	40.81%	40.90%	40.92%

Table 3. Statistics of KOREF genome assemblies using ONT PromethION and PacBio Sequel sequencing with Hi-C mapping information

	**ONT PromethION R9.4.1**		**PacBio Sequel**	
	**27× assembly with Hi-C**	**64× assembly with Hi-C**	**30× assembly with Hi-C**	**62× assembly with Hi-C**
**Scaffolds No.**	2,313	3,179	1,476	2,139
**Total length (bp)**	2,757,776,303	2,827,900,542	2,801,450,512	2,815,594,432
**N50 (bp)**	32,758,624	56,457,651	38,113,117	59,361,327
**Max scaffold length (bp)**	120,666,262	175,227,974	126,818,544	174,360,016
**Gap**	0.02%	0.01%	0.02%	0.01%
**GC content**	40.82%	40.81%	40.90%	40.90%

Table 4. Statistics of KOREF genome assembly assessment using BUSCO and accuracy comparison

	ONT PromethION R9.4.1				PacBio Sequel			
**BUSCO assessment**	**27× assembly**	**64× assembly**	**27× assembly with Hi-C**	**64× assembly with Hi-C**	**30× assembly**	**62× assembly**	**30×assembly with Hi-C**	**62× assembly with Hi-C**
**Complete**	92.5%	92.7%	92.6%	94.0%	93.8%	93.9%	93.8%	93.5%
**Complete and single-copy**	91.8%	91.6%	91.9%	93.2%	93.0%	93.1%	93.0%	92.7%
**Complete and duplicated**	0.7%	1.1%	0.7%	0.8%	0.8%	0.8%	0.8%	0.8%
**Fragmented**	3.1%	3.7%	3.2%	3.1%	3.0%	2.9%	3.0%	3.1%
**Missing**	4.4%	3.6%	4.2%	2.9%	3.2%	3.2%	3.2%	3.4%
**Accuracy comparison***	99.78%	99.73%	99.78%	99.73%	99.83%	99.79%	99.86%	99.80%

***** Compared with KOREF_S, the single assembly of KOREF

Figure 1

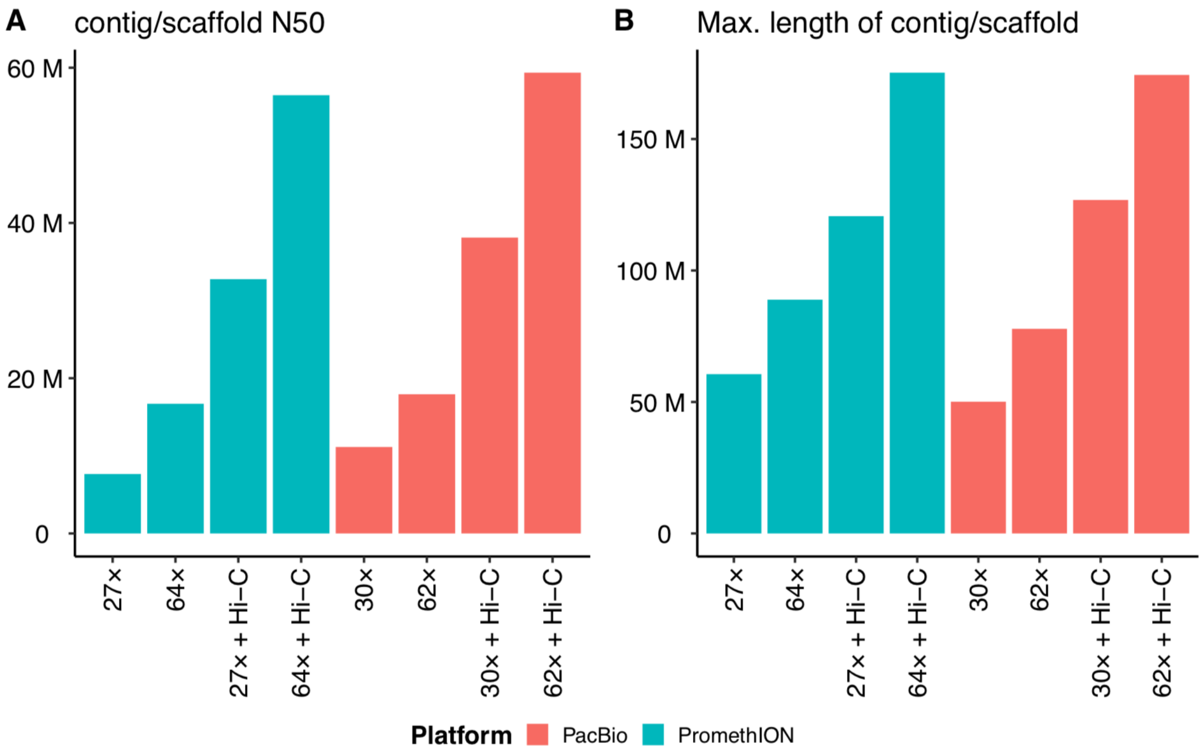
A
contig/scaffold N50
60 M
40 M
20 M
0
27×
64×
27× + Hi–C
64× + Hi–C
30×
62×
30× + Hi–C
62× + Hi–C
B
Max. length of contig/scaffold
150 M
100 M
50 M
0
27×
64×
27× + Hi–C
64× + Hi–C
30×
62×
30× + Hi–C
62× + Hi–C
Platform
PacBio
PromethION
