# Supplementary material for: Chromosome-scale assembly comparison of the Korean Reference Genome KOREF from PromethION and PacBio with Hi-C mapping information
Source: Gigascience. 2019 Dec 3;8(12):giz125. doi: 10.1093/gigascience/giz125 (PMC6889754; doi:10.1093/gigascience/giz125)
Supplement: giz125_Response_to_Reviewer_Comments_Original_Submission [file giz125_response_to_reviewer_comments_original_submission.pdf]

Author's Response To Reviewer Comments

Close

Reviewer reports:

Reviewer #1:
Kim et al. generated multiple de novo assemblies of the Korean Reference Genome KOREF using nanopore and PacBio sequence data. These assemblies were polished with available short read data and further scaffolded with HiC data. These should be useful assemblies for the scientific community. All the data appears proper. I have a few issues that I think need to be resolved below:

1. Page 9, 7-16 talks about scaffold N50s, those same N50 lengths are referred to as contigs in the abstract. Which is it scaffolds or contigs? These two things are not the same.

 You are right. To clarify: We used the concept of contigs and scaffolds as below. A contig is a contiguous genomic sequence without gaps in which the order of bases is known to a high confidence level. Mostly contigs are composed of overlapped reads from short or long read sequencing.
A scaffold is a portion of the genome sequence reconstructed from long range mated-pair short reads or the long-range mapping information such as Hi-C and BioNano data.
Therefore, the result from our assemblies with only long read sequencing was called as 'contigs' and the result from assemblies with Hi-C was denoted as 'scaffolds'.
 To be clear, we changed the terms (contig or scaffold) of page 9, 7 – 16, accordingly.

2. Table 2 and Figure 1. Scaffolds and contigs are mixed here again. Why? This is confusing. These two things should be separated, they aren't the same. Perhaps with nanopore/Pacbio data only there's no difference between contig and scaffold length. However, the addition of Hi-C data would be expected to increase scaffold length but have little effect on contig length.

 Good point. A new table with separated results from contigs and scaffolds is available (Table 2 and 3). We hope that this can be clear. We revised the legend of the Figure1.

Minor/interesting analyses:
1. Perhaps you are already doing this for another manuscript, but I think it would be interesting to compare between the different assemblies to see what regions are present/missing in nanopore versus PacBio. Was diploid assembly possible?

 Interesting point. Thank you. Yes, we have assembled haplotype-resolved assemblies using the trio-binning method with KOREF_S (single individual reference). We are preparing another manuscript for these results and other in-depth analyses to see if PacBio and Promethion have segment differences in the assemblies. It is out of the scope of this paper. Although, it won't be included in the current paper, below is a preliminary alignment difference representation.

https://github.com/macarima/KOREF_PromethION_paper/blob/master/Supplementary_figures/images/Figure1-1.png

https://github.com/macarima/KOREF_PromethION_paper/blob/master/Supplementary_figures/images/Figure1-2.png

 We also performed analysis of structural variation with assemblies. The patterns looked very similar (SVs from PromethION showed similar results to PacBio's). The analysis was performed using Nucmer program from Mummer (https://github.com/mummer4/mummer) and Assemblytics v 1.0 (https://doi.org/10.1093/bioinformatics/btw369). These are not included in the revision.

https://github.com/macarima/KOREF_PromethION_paper/blob/master/Supplementary_figures/images/Figure2-1.png

https://github.com/macarima/KOREF_PromethION_paper/blob/master/Supplementary_figures/images/Figure2-2.png

2. If you try to call variants off of this assembly how well does it perform, perhaps since it's not diploid this won't work well.

 It is another interesting research point. Yes, this is not a phased diploid assembly. It is supposed to be difficult to call variants efficiently without phasing information. We did not attempt to call.

Reviewer #2:
The authors compare assemblies from two different long read sequencing datasets (PacBio CLR and Oxford Nanopore Promethion) on the same Korean reference sample. These results are well-written, interesting, and useful to the community, though it would be good to add caveats to some of the conclusions given these quickly evolving technologies. In particular, it would be useful to discuss how these compare to the new PacBio CCS/HiFI assemblies. I also have a few suggestions below:

1. Abstract: "PacBio's SMRT sequencing is expensive for a full human genome assembly and costs over 40,000 USD for 30× coverage as of 2019"
- It's probably best to either make this less precise or say "early 2019" because my understanding is that the Sequel II now can give ~30x CCS coverage for $10-15k.

 We agree. According to the reviewer's suggestion, we changed "2019" to "early 2019". Sequel II platform seems to show good performance with high accuracy (99.95% accuracy, https://doi.org/10.1101/635037) and is more cost-effective than Sequel.
 About contiguity, N50s between CCS and CLS assembly look almost the same. Sequel II can be benchmarked with PromethION R10 flowcell data which will be released soon (~Sep. 2019). PromethION R10 seems to have high accuracy as well (99.999% from the Oxford nanopore claim) like Sequel II (https://nanoporetech.com/about-us/news/new-r10-nanopore-released-early-access). When the new flowcells are released (R10), we will compare them and perhaps report in another manuscript.

2. Abstract: "The pore-based PromethION approach provides a good quality chromosome-scale human genome assembly at a low cost with long maximum contig and scaffold lengths and is more cost-effective than PacBio at comparable quality measurements"
- It would be good to discuss accuracy of the assemblies in addition to N50 metrics in the abstract, and ideally do a deeper analysis of accuracy.

 It is an important point and of great interest, too. As it would take too much cross-validation and even more sequencing with short-reads, we cannot give a really high quality accuracy measurement here. However, we have performed alignment and calculated the accuracy with highly accurate KOREF_S assembly which has been assembled with both Illumina short reads and PacBio RS2 in Table 4 (Accuracy comparison section). We hope this can give some approximation for the current accuracy.
 Again, when the new R10 Flowcell from ONT is released, we could do much more meaningful accuracy comparison while this manuscript focuses on cost-effectiveness of the two major single molecular long read sequencing platforms.

3. Page 9: "Long read assemblies from 27× and 64× PromethION sequencing yielded total assembly sizes of 2,757 Mbp and 2,827 Mbp, with scaffold N50s of 7.6 Mbp and 16.7 Mbp, respectively (Table 2). Assemblies from PacBio sequencing at 30× and 62× coverage yielded the total assembly sizes of 2,800 Mbp and 2,815 Mbp, with scaffold N50s of 11.1 Mbp and 17.9 Mbp, respectively."
-Before HiC, are the contig and scaffold N50's the same? If so, it would be good to state this explicitly

 Thank you. It was not clear. For constructing chromosome scaled scaffolds, Hi-C map assembly was performed

with ‘contigs’ from de novo assemblies with PromethION or PacBio reads. To make it clear, we have now divided ‘Table 2’ to Table 2 (statistics from contigs) and 3 (statistics from scaffolds).

4. Table 1: Are the PacBio read lengths the "sub-read" lengths, i.e. the lengths after breaking raw reads at the SMRT-bell adaptor sequences? If not, the sub-read statistics should also be given.

 Thank you. Yes, they are the “sub-read” lengths.

5. It would be useful for the authors to discuss how this compares with the new PacBio CCS/HiFi method in these papers: https://doi.org/10.1101/635037and https://doi.org/10.1101/519025

 Thank you for your advice. We have read the mentioned papers and have plans to produce CCS reads from Sequel II platform with the KOREF cell lines (KOREF_S has publically available cell-lines). In the near future, we will compare the PacBio CCS results with the one of PromethION R10.
 For comparison between KOREF PromethION assemblies and CHM3 PacBio Sequel II assembly with the identical condition, we performed CHM3 assembly with wtdbg2 v2.3. Parameters which we used and other information could be found in the github page (https://github.com/macarima/KOREF_PromethION_paper). In terms of N50, the PacBio Sequel II assembly at 25× coverage yielded 1.9-fold and 0.8-fold longer N50s compared with the PromethION assemblies at 27× and 64× coverage, respectively. When we compared the longest contigs, the PacBio Sequel II assembly yielded 1.2-fold and 0.8-fold length increase compared with the PromethION assemblies at 27× and 64× coverage, respectively (Table S1).

https://github.com/macarima/KOREF_PromethION_paper/blob/master/Supplementary_figures/images/TableS1.png

6. I could not find the methods for base calling promethion data, which are important because the ONT base caller is quickly improving.

 Good point. Thank you. Basecalling PromethION data was conducted using guppy v2.1.3 with the Transducer model. We added this information to the manuscript.

To reviewers,

All figures and tables are available on the github page (https://github.com/macarima/KOREF_PromethION_paper/tree/master/Supplementary_figures).
Thank you.

Close
