# Supplementary material for: Chromosome-scale assembly comparison of the Korean Reference Genome KOREF from PromethION and PacBio with Hi-C mapping information
Source: Gigascience. 2019 Dec 3;8(12):giz125. doi: 10.1093/gigascience/giz125 (PMC6889754; doi:10.1093/gigascience/giz125)
Supplement: giz125_Reviewer_1_Report_Original_Submission — Brock Peters, Ph.D. -- 7/15/2019 Reviewed [file giz125_reviewer_1_report_original_submission.pdf]

Reviewer Report

Title: Chromosome-scale assembly comparison of the Korean Reference Genome KOREF from PromethION and PacBio with Hi-C mapping information

Version: Original Submission **Date:** 7/15/2019

Reviewer name: Brock Peters, Ph.D.

Reviewer Comments to Author:

Kim et al. generated multiple de novo assemblies of the Korean Reference Genome KOREF using nanopore and PacBio sequence data. These assemblies were polished with available short read data and further scaffolded with HiC data. These should be useful assemblies for the scientific community. All the data appears proper. I have a few issues that I think need to be resolved below:

1. Page 9, 7-16 talks about scaffold N50s, those same N50 lengths are referred to as contigs in the abstract. Which is it scaffolds or contigs? These two things are not the same.
2. Table 2 and Figure 1. Scaffolds and contigs are mixed here again. Why? This is confusing. These two things should be separated, they aren't the same. Perhaps with nanopore/Pacbio data only there's no difference between contig and scaffold length. However, the addition of HiC data would be expected to increase scaffold length but have little effect on contig length.

Minor/interesting analyses:

Perhaps you are already doing this for another manuscript, but I think it would be interesting to compare between the different assemblies to see what regions are present/missing in nanopore versus PacBio.

Was diploid assembly possible?

If you try to call variants off of this assembly how well does it perform, perhaps since it's not diploid this won't work well.

Level of Interest

Please indicate how interesting you found the manuscript: Choose an item.

Quality of Written English

Please indicate the quality of language in the manuscript: Choose an item.

Declaration of Competing Interests

Please complete a declaration of competing interests, considering the following questions:

- Have you in the past five years received reimbursements, fees, funding, or salary from an organisation that may in any way gain or lose financially from the publication of this manuscript, either now or in the future?
- Do you hold any stocks or shares in an organisation that may in any way gain or lose financially from the publication of this manuscript, either now or in the future?
- Do you hold or are you currently applying for any patents relating to the content of the manuscript?
- Have you received reimbursements, fees, funding, or salary from an organization that holds or has applied for patents relating to the content of the manuscript?
- Do you have any other financial competing interests?
- Do you have any non-financial competing interests in relation to this paper?

If you can answer no to all of the above, write 'I declare that I have no competing interests' below. If your reply is yes to any, please give details below.

I declare that I have no competing interests

I agree to the open peer review policy of the journal. I understand that my name will be included on my report to the authors and, if the manuscript is accepted for publication, my named report including any attachments I upload will be posted on the website along with the authors' responses. I agree for my report to be made available under an Open Access Creative Commons CC-BY license (http://creativecommons.org/licenses/by/4.0/). I understand that any comments which I do not wish to be included in my named report can be included as confidential comments to the editors, which will not be published.

Choose an item.

To further support our reviewers, we have joined with Publons, where you can gain additional credit to further highlight your hard work (see: https://publons.com/journal/530/gigascience). On publication of this paper, your review will be automatically added to Publons, you can then choose whether or not to claim your Publons credit. I understand this statement.

Yes Choose an item.
