# Supplementary material for: Chromosome-scale assembly comparison of the Korean Reference Genome KOREF from PromethION and PacBio with Hi-C mapping information
Source: Gigascience. 2019 Dec 3;8(12):giz125. doi: 10.1093/gigascience/giz125 (PMC6889754; doi:10.1093/gigascience/giz125)
Supplement: giz125_Reviewer_2_Report_Original_Submission — Justin Zook -- 7/18/2019 Reviewed [file giz125_reviewer_2_report_original_submission.pdf]

Reviewer Report

Title: Chromosome-scale assembly comparison of the Korean Reference Genome KOREF from PromethION and PacBio with Hi-C mapping information

Version: Original Submission **Date:** 7/18/2019

Reviewer name: Justin Zook

Reviewer Comments to Author:

The authors compare assemblies from two different long read sequencing datasets (PacBio CLR and Oxford Nanopore Promethion) on the same Korean reference sample. These results are well-written, interesting, and useful to the community, though it would be good to add caveats to some of the conclusions given these quickly evolving technologies. In particular, it would be useful to discuss how these compare to the new PacBio CCS/HiFI assemblies. I also have a few suggestions below:
1. Abstract: "PacBio's SMRT sequencing is expensive for a full human genome assembly and costs over 40,000 USD for 30Ã— coverage as of 2019"
- It's probably best to either make this less precise or say "early 2019" because my understanding is that the Sequel II now can give ~30x CCS coverage for $10-15k
2. Abstract: "The pore-based PromethION approach provides a good quality chromosome-scale human genome assembly at a low cost with long maximum contig and scaffold lengths and is more cost-effective than PacBio at comparable quality measurements"
- It would be good to discuss accuracy of the assemblies in addition to N50 metrics in the abstract, and ideally do a deeper analysis of accuracy.
3. Page 9: "Long read assemblies from 27Ã— and 64Ã— PromethION sequencing yielded total assembly sizes of 2,757 Mbp and 2,827 Mbp, with scaffold N50s of 7.6 Mbp and 16.7 Mbp, respectively (Table 2). Assemblies from PacBio sequencing at 30Ã— and 62Ã— coverage yielded the total assembly sizes of 2,800 Mbp and 2,815 Mbp, with scaffold N50s of 11.1 Mbp and 17.9 Mbp, respectively."
- Before HiC, are the contig and scaffold N50's the same? If so, it would be good to state this explicitly
4. Table 1: Are the PacBio read lengths the "sub-read" lengths, i.e. the lengths after breaking raw reads at the SMRT-bell adaptor sequences? If not, the sub-read statistics should also be given.
5. It would be useful for the authors to discuss how this compares with the new PacBio CCS/HiFi method in these papers: https://doi.org/10.1101/635037 and https://doi.org/10.1101/519025
6. I could not find the methods for base calling promethion data, which are important because the ONT base caller is quickly improving.

Level of Interest

Please indicate how interesting you found the manuscript: Choose an item.

Quality of Written English

Please indicate the quality of language in the manuscript: Choose an item.

Declaration of Competing Interests

Please complete a declaration of competing interests, considering the following questions:

- Have you in the past five years received reimbursements, fees, funding, or salary from an organisation that may in any way gain or lose financially from the publication of this manuscript, either now or in the future?
- Do you hold any stocks or shares in an organisation that may in any way gain or lose financially from the publication of this manuscript, either now or in the future?
- Do you hold or are you currently applying for any patents relating to the content of the manuscript?
- Have you received reimbursements, fees, funding, or salary from an organization that holds or has applied for patents relating to the content of the manuscript?
- Do you have any other financial competing interests?
- Do you have any non-financial competing interests in relation to this paper?

If you can answer no to all of the above, write 'I declare that I have no competing interests' below. If your reply is yes to any, please give details below.

I declare that I have no competing interests

I agree to the open peer review policy of the journal. I understand that my name will be included on my report to the authors and, if the manuscript is accepted for publication, my named report including any attachments I upload will be posted on the website along with the authors' responses. I agree for my report to be made available under an Open Access Creative Commons CC-BY license (http://creativecommons.org/licenses/by/4.0/). I understand that any comments which I do not wish to be included in my named report can be included as confidential comments to the editors, which will not be published.

Choose an item.

To further support our reviewers, we have joined with Publons, where you can gain additional credit to further highlight your hard work (see: https://publons.com/journal/530/gigascience). On publication of this paper, your review will be automatically added to Publons, you can then choose whether or not to claim your Publons credit. I understand this statement.

Yes Choose an item.
